# Reporter gene-expressing bone marrow-derived stromal cells are immune-tolerated following implantation in the central nervous system of syngeneic immunocompetent mice

**DOI:** 10.1186/1472-6750-9-1

**Published:** 2009-01-07

**Authors:** Irene Bergwerf, Nathalie De Vocht, Bart Tambuyzer, Jacob Verschueren, Kristien Reekmans, Jasmijn Daans, Abdelilah Ibrahimi, Viggo Van Tendeloo, Shyama Chatterjee, Herman Goossens, Philippe G Jorens, Veerle Baekelandt, Dirk Ysebaert, Eric Van Marck, Zwi N Berneman, Annemie Van Der Linden, Peter Ponsaerts

**Affiliations:** 1 Laboratory of Experimental Hematology, University of Antwerp, Antwerp, Belgium; 2 Vaccine and Infectious Disease Institute, University of Antwerp, Antwerp, Belgium; 3 Bio-Imaging Laboratory, University of Antwerp, Antwerp, Belgium; 4 Laboratory for Molecular Virology & Gene Therapy, Katholieke Universiteit Leuven, Leuven, Belgium; 5 Laboratory of Pathology, University of Antwerp, Antwerp, Belgium; 6 Clinical Pharmacotherapy, University of Antwerp, Antwerp, Belgium; 7 Laboratory for Neurobiology & Gene Therapy, Katholieke Universiteit Leuven, Leuven, Belgium; 8 Laboratory of Experimental Surgery, University of Antwerp, Antwerp, Belgium; 9 Centre for Cellular Therapy and Regenerative Medicine (CCRG), Antwerp University Hospital, Antwerp, Belgium; 10 Molecular Small Animal Imaging Centre (Mosaic), Katholieke Universiteit Leuven, Leuven, Belgium

## Abstract

**Background:**

Cell transplantation is likely to become an important therapeutic tool for the treatment of various traumatic and ischemic injuries to the central nervous system (CNS). However, in many pre-clinical cell therapy studies, reporter gene-assisted imaging of cellular implants in the CNS and potential reporter gene and/or cell-based immunogenicity, still remain challenging research topics.

**Results:**

In this study, we performed cell implantation experiments in the CNS of immunocompetent mice using autologous (syngeneic) luciferase-expressing bone marrow-derived stromal cells (BMSC-Luc) cultured from ROSA26-L-S-L-Luciferase transgenic mice, and BMSC-Luc genetically modified using a lentivirus encoding the enhanced green fluorescence protein (eGFP) and the puromycin resistance gene (Pac) (BMSC-Luc/eGFP/Pac). Both reporter gene-modified BMSC populations displayed high engraftment capacity in the CNS of immunocompetent mice, despite potential immunogenicity of introduced reporter proteins, as demonstrated by real-time bioluminescence imaging (BLI) and histological analysis at different time-points post-implantation. In contrast, both BMSC-Luc and BMSC-Luc/eGFP/Pac did not survive upon intramuscular cell implantation, as demonstrated by real-time BLI at different time-points post-implantation. In addition, ELISPOT analysis demonstrated the induction of IFN-γ-producing CD8+ T-cells upon intramuscular cell implantation, but not upon intracerebral cell implantation, indicating that BMSC-Luc and BMSC-Luc/eGFP/Pac are immune-tolerated in the CNS. However, in our experimental transplantation model, results also indicated that reporter gene-specific immune-reactive T-cell responses were not the main contributors to the immunological rejection of BMSC-Luc or BMSC-Luc/eGFP/Pac upon intramuscular cell implantation.

**Conclusion:**

We here demonstrate that reporter gene-modified BMSC derived from ROSA26-L-S-L-Luciferase transgenic mice are immune-tolerated upon implantation in the CNS of syngeneic immunocompetent mice, providing a research model for studying survival and localisation of autologous BMSC implants in the CNS by real-time BLI and/or histological analysis in the absence of immunosuppressive therapy.

## Background

Cell transplantation is likely to become an important therapeutic tool for the treatment of various traumatic and ischemic injuries to the central nervous system (CNS). While injuries to the CNS have been shown to trigger neurogenesis from resident neural stem cells, these endogenous self-repair mechanisms are insufficient to induce full functional recovery [1,2]. Therefore, it is clear that additional therapies, like cell transplantation, might be needed to further enhance restoration of brain function following primary (e.g. impact, stroke) and secondary (e.g. inflammation) injury to the CNS. Although many studies aim to replace necrotic or dysfunctional neural tissue directly by implantation of stem cells, only modest functional recovery following injury has been observed until now [3-5]. A more realistic aim for stem cell therapy to restore injuries to the CNS might be the implantation of genetically modified stem cell populations in order to produce neurotrophic factors (like BDNF, NT3 or GDNF), with the potential to enhance survival of existing neurons and endogenous neuroregeneration [6,7]. This approach is currently well-described by several research groups including ours [8-11]. For these studies, most ideally one should be able to non-invasively visualise and localise stem cell implants in the brain of living animals at different time-points. For this purpose, both bioluminescence imaging (BLI) and magnetic resonance imaging (MRI) have been proposed as suitable non-invasive methodologies for the follow-up of cell implants in the CNS of rodents [12-15]. While images created by MRI have a high spatial resolution, cells need to be loaded with contrast agents, like super paramagnetic iron oxides (SPIO), which might display some toxicity towards the implanted cells and surrounding tissue. Another disadvantage of these contrast agents is leakage out of necrotic cells and uptake by endogenous cells, which might result in false identification of cell implant survival and localisation. In contrast, for generating images by BLI, cell implants need to express the luciferase reporter protein, which, following administration of the substrate luciferin, can produce light through an ATP-dependent enzymatic oxidation of luciferin. Therefore, despite the lower special resolution than MRI, BLI visualises only viable cell implants, which makes BLI one of the most valuable research techniques in order to monitor survival of cell implants non-invasively. One potential drawback of BLI is the need for genetic modification of cell populations with the Luciferase reporter gene. While it has been clearly documented that the enhanced green fluorescent protein (eGFP), which is currently the main reporter gene for histological analysis of cell implants, is a strong immunogenic antigen and requires the need for immune suppressive therapy during cell implantation experiments in non-CNS tissues, it is at the moment rather unclear whether the eGFP or luciferase reporter proteins are tolerated by the immune system following cell implantation in the CNS of immune competent animals [16-19].

## Methods

### Animals

Homozygous ROSA26-L-S-L-Luciferase transgenic mice (FVB background) were obtained via Jackson Laboratories (strain 005125) and further bred in the specific pathogen free animal facility of the University of Antwerp [20]. Male offspring (n = 90) were used for bone marrow-derived stromal cell (BMSC) culture, cell implantation experiments and/or ELISPOT analysis. For all experiments, mice were kept in normal day-night cycle (12/12) with free access to food and water. All experimental procedures were approved by the Ethics Committee for Animal Experiments of the University of Antwerp (approval no. 2006/36).

### Establishment and maintenance of primary BMSC cultures

BMSC were cultured from male ROSA26-L-S-L-Luciferase transgenic mice following a protocol previously described by Peister et al. [21]. Briefly, bone marrow was flushed from tibia and femurs of 3-week old ROSA26-L-S-L-Luciferase mice. Next, harvested bone marrow was washed twice with phosphate-buffered saline (PBS) and the total cell population obtained was plated in a T75 culture flask (one flask per mouse) in 20 ml 'complete isolation medium' (CIM), consisting of RPMI-1640 medium (Invitrogen) supplemented with 8% horse serum (HS, Invitrogen), 8% fetal calf serum (FCS, Hyclone), 100 U/ml penicillin (Invitrogen), 100 mg/ml streptomycin (Invitrogen), and 1.25 mg/ml amphotericin B (Invitrogen). Following 24 hours of culture, non-adherent cells were removed and 20 ml fresh CIM was added to the cultures. For a period of two weeks, CIM was replaced every 3 to 4 days. Next, cultured cells were harvested using trypsin-EDTA (Invitrogen) treatment and replated in a new T75 culture flask in 20 ml CIM. Stromal cell outgrowth in this culture was termed passage 1 and further expanded in 'complete expansion medium' (CEM), consisting of Iscove modified Dulbecco's medium (IMDM, Cambrex) supplemented with 8% FCS, 8% HS, 100 U/ml penicillin, 100 mg/ml streptomycin and 1.25 mg/ml amphotericin B. For routine cell culture, BMSC cultures were split 1:3 every 5 to 7 days. In addition, clonal cultures of luciferase-expressing stromal cells were obtained by limiting dilution.

### Flow cytometry

Immunophenotyping of BMSC cultures derived from ROSA26-L-S-L-Luciferase transgenic mice was performed using the following monoclonal antibodies: fluorescein-isothiocyanate (FITC)-labelled anti-mouse CD31 (eBioscience, 11/0311-82), FITC-labelled anti-mouse CD106 (eBioscience, 11/1081-82), FITC-labelled anti-mouse CD117 (eBioscience, 11/1171-82), FITC-labelled anti-mouse Sca-1 (eBioscience, 11/5981-82), FITC-labelled anti-mouse MHC-I (Becton Dickinson, 5553570), phycoerythrin (PE)-labeled anti-mouse CD45 (Becton Dickinson, 553081), and PE-labelled anti-mouse MHC-II (eBioscience, 12/5321/82). Immunostaining for A2B5 was performed using an unconjugated mouse-anti-mouse A2B5 monoclonal antibody (Chemicon, MAB312R) followed by staining with PE-labelled rat-anti-mouse secondary antibody (Jackson Immunoresearch, 115-116-075). Before staining, harvested cells were washed twice with PBS supplemented with 1% FCS (designated as PBS*) and resuspended in PBS* at a concentration of 5 × 10^5 ^cells/ml. For antibody staining, 1 μg of antibody was added to 100 μl of cell suspension for 30 min at 4°C. Following incubation, cells were washed once with PBS*, resuspended in 1 mL PBS*, and analysed using an Epics XL-MCL analytical flow cytometer (Beckman Coulter). For determination of eGFP transgene expression, harvested eGFP mRNA-electroporated or lentivirus-transduced BMSC cultures were washed once with PBS, resuspended in PBS and directly analysed using an Epics XL-MCL analytical flow cytometer. Cell viability was assessed through addition of GelRed (1× final concentration, Biotum) to the cell suspension immediately before flow cytometric analysis. At least 10,000 cells per sample were analysed per sample and flow cytometry data were analysed using FlowJo software.

### Messenger RNA electroporation

Messenger (m)RNA encoding the enhanced green fluorescent protein (eGFP) and the Cre recombinase protein was prepared as described previously [22,23]. Prior to electroporation of BMSC populations, cells were washed twice with serum-free OptiMem medium (Invitrogen) and resuspended at a final concentration of 5–10 × 10^6 ^cells/ml in serum-free OptiMem medium. Subsequently, 200 μl of the cell suspension was mixed with 20 μg of mRNA and electroporated in a 4 mm electroporation cuvette at 300V and 150 μF using a Gene Pulser Xcell electroporation device (Bio-Rad). After electroporation, fresh complete medium was added to the cell suspension and cells were further cultured as described above.

### In vitro bioluminescence assay

Luciferase activity in cultured BMSC, BMSC-Luc and BMSC-Luc/eGFP/Pac cell populations (1 × 10^5 ^cells per assay) was measured using the commercial Bright-Glo luciferase assay system (Promega), according to the manufacturer's instructions.

### Lentiviral construction

Construction of the pCHMWS-eGFP-IRES-Pac vector was performed in two consecutive steps using standard cloning techniques. First, the puromycin resistance gene (Pac) was inserted downstream of an IRES element and the resulting IRES-Pac clone was amplified by PCR and cloned after the eGFP in the pCHMWS-eGFP vector [24].

### Lentiviral transduction

Lentiviral vector production was performed as described earlier by Geraerts et al., with minor modifications [25]. Filtered vector particles were concentrated using Vivaspin 15 columns (Vivascience, Hannover, Germany), aliquoted and stored at -80°C. For transduction experiments, cells were seeded in a 24-well plate at 50,000 cells per well. The next day, cells were transduced with vector expressing the eGFP-IRES-Pac cassette (2.86 10^5^pg p24/well) in CEM medium. After 48 hrs of incubation, the vectors were washed from the cells and medium was replaced. Cells were subcultured at least 4 times and transduction efficiency was determined by flow cytometry. In addition, a clonal line was obtained by limiting dilution for use in further cell implantation experiments.

### Cell preparation for implantation experiments

Following harvesting of BMSC-Luc and BMSC-Luc/eGFP/Pac cell populations via trypsin/EDTA treatment, cells were washed twice with PBS. Next, cells (mean viability of cell populations was 90–95%) were resuspended at a concentration of 100 × 10^6 ^cells/mL in PBS for intracerebral cell implantation or at a concentration of 5 × 10^6 ^cells/mL in PBS for intramuscular cell implantation. Cell preparations were kept on ice until intracerebral cell implantation.

### Cell transplantation experiments

For cell implantation in the CNS, mice were anaesthetized by an intraperitoneal injection of a ketamin (80 mg/kg) + xylazin (16 mg/kg) mixture and placed in a stereotactic frame. Next, a midline scalp incision was made and a hole was drilled in the skull using a dental drill burr at an equal distance between RCS and lambda and at 2 mm on the right side of the midline. Thereafter, an automatic micro-injector pump (kdScientific) with a 10 μl Hamilton Syringe was positioned above the exposed dura. A 30-gauge needle (Hamilton), attached to the syringe, was stereotactically placed through the intact dura to a depth of 2 mm. After 2 minutes of pressure equilibration, 2 × 10^5 ^BMSC-Luc or BMSC-Luc/eGFP/Pac in 2 μl PBS were injected at 0.7 μl/min. The needle was retracted after another 3 minutes to allow pressure equilibration and to prevent backflow of the injected cell suspension. Next, the skin was sutured, a 0.9% NaCl solution was administered subcutaneously in order to prevent dehydration and mice were placed under a heating lamp to recover. For intramuscular cell injection, mice were anaesthetized in an induction chamber using an isoflurane (3%) + N_2 _(1 L/min) + O_2 _(0,5 L/min) gas mixture. Directly thereafter, 5 × 10^5 ^BMSC-Luc or BMSC-Luc/eGFP/Pac in 100 μl PBS were injected in the right pelvic limb muscles.

### In vivo bioluminescence imaging

At different time points between day 1 and week 4 after cell implantation, mice were analysed by real-time *in vivo *bioluminescence imaging (BLI) in order to determine the presence or absence of viable cell implants in the CNS. For this, mice were anaesthetized by intraperitoneal injection of a ketamin (80 mg/kg) + xylazin (16 mg/kg) mixture, followed by an intraperitoneal (brain BLI) or intravenous (muscle BLI) injection of D-luciferin (150 mg/kg body weight dissolved in PBS, Synchem). Immediately after luciferin administration, mice were imaged for 20 minutes using an *in vivo *real-time φ-imager system (Biospace). At the end of every acquisition a photographic image was obtained. The data were analysed with Photovision software, which superimposes the bioluminescence signal on the photographic image. The most intense bioluminescence signal detected is shown in red, while the weakest signal is shown in blue.

### Brain dissection for histological analysis

At week 1 or week 3 post-implantation, mice were deeply anaesthetized in an induction chamber by inhalation of an isofluorane (4%), oxygen (0,5 L/min) and nitrogen (1 L/min) mixture for 2 minutes, followed by cervical dislocation. Whole brains were surgically removed and fixed in 4% paraformaldehyde for 2 hours.

### Histological analysis

Fixed brains were dehydrated in sucrose gradients (5%, 10% and 20%), frozen in liquid nitrogen and stored at -80°C until further processing. Consecutive 10 μm-thick cryosections were cut using a Microm HM5000 cryostat and stained with haematoxylin-eosin (HE) to locate the transplantation site. Further immunohistochemical analysis was performed using a biotin-labeled anti-mouse Sca-1 antibody (eBioscience 13-5981-85) for BMSC identification, and a biotin-labeled anti-mouse CD11b antibody (eBioscience 13-0112-85) for detection of activated microglia at the site of cell implantation. In brief, slides were rinsed with a washing buffer and endogenous peroxidase was blocked following 30 min incubation with methanol containing 1% hydrogen peroxide. Next, slides were washed with water and washing buffer, followed by incubation with normal rat serum (Jackson Immuno Research 012-000-120) for 1 hour at room temperature. Subsequently, slides were incubated for 3 hours with the biotin-labeled primary antibody at room temperature. Following this, slides were rinsed with washing buffer, and incubated for 1 hour at room temperature with a streptavidin-horse-radish-peroxidase complex (Dako 00032671). Visualization for all slides was carried out after staining with diaminobenzidine (DAB, Dako), according to manufacturer's instructions, and nuclei were counterstained with Carazzi's haematoxylin. Bright-field immunohistochemical analysis was done using an Olympus Bx41 microscope equipped with an Olympus DP50 camera. Olympus DP Software was used for image collection.

### ELISPOT analysis

A murine IFN-γ ELISPOT assay (Diaclone, 862.031.010.S) was performed according to manufacturer's instructions. In brief, spleens were dissected from cell-transplanted ROSA26-L-S-L-Luciferase transgenic mice (both intramuscular and intracerebral) at 2 weeks post-injection. Next, after dissociation of the spleens over a 100 μm nylon filter, mononuclear cells were enriched following a density-based centrifugation step (Ficoll-Paque Plus, GE Healthcare). Magnetic isolation of CD8+ T-cells was done using anti-CD8 MACS MicroBeads (Miltenyi Biotec, 130-049-401), according to the manufacturer's instructions. Isolated CD8+ T-cells (= responder cells) were plated on ELISPOT plates at 1 × 10^5 ^cells/well in IMDM supplemented with 10% FBS, penicillin/streptomycin and amphotericin B. Cells were then cultured for 16 hours either: (i) un-stimulated, (ii) stimulated with 1 × 10^4 ^parental BMSC (= non stimulator cells), or (III) stimulated with 1 × 10^4 ^BMSC-Luc or BMSC-Luc/eGFP/Pac (= stimulator cells). All experiments were performed in quadruplicate per mouse. The ELISPOT plates were analysed using an AID ELISPOT Reader (Autoimmun Diagnostika GmbH). Data are presented as IFN-γ spot-forming cells (SFC) per 1 × 10^5 ^CD8+ responder T-cells.

### Statistical analysis

Results are expressed as mean ± standard deviation. Comparisons were validated using Student's t-test. A p-value < 0.01 was considered to be statistically significant.

## Results

### Culture and characterisation of a clonal luciferase-expressing bone marrow-derived stromal cell line from ROSA26-L-S-L-Luciferase transgenic mice

**Figure 1:**
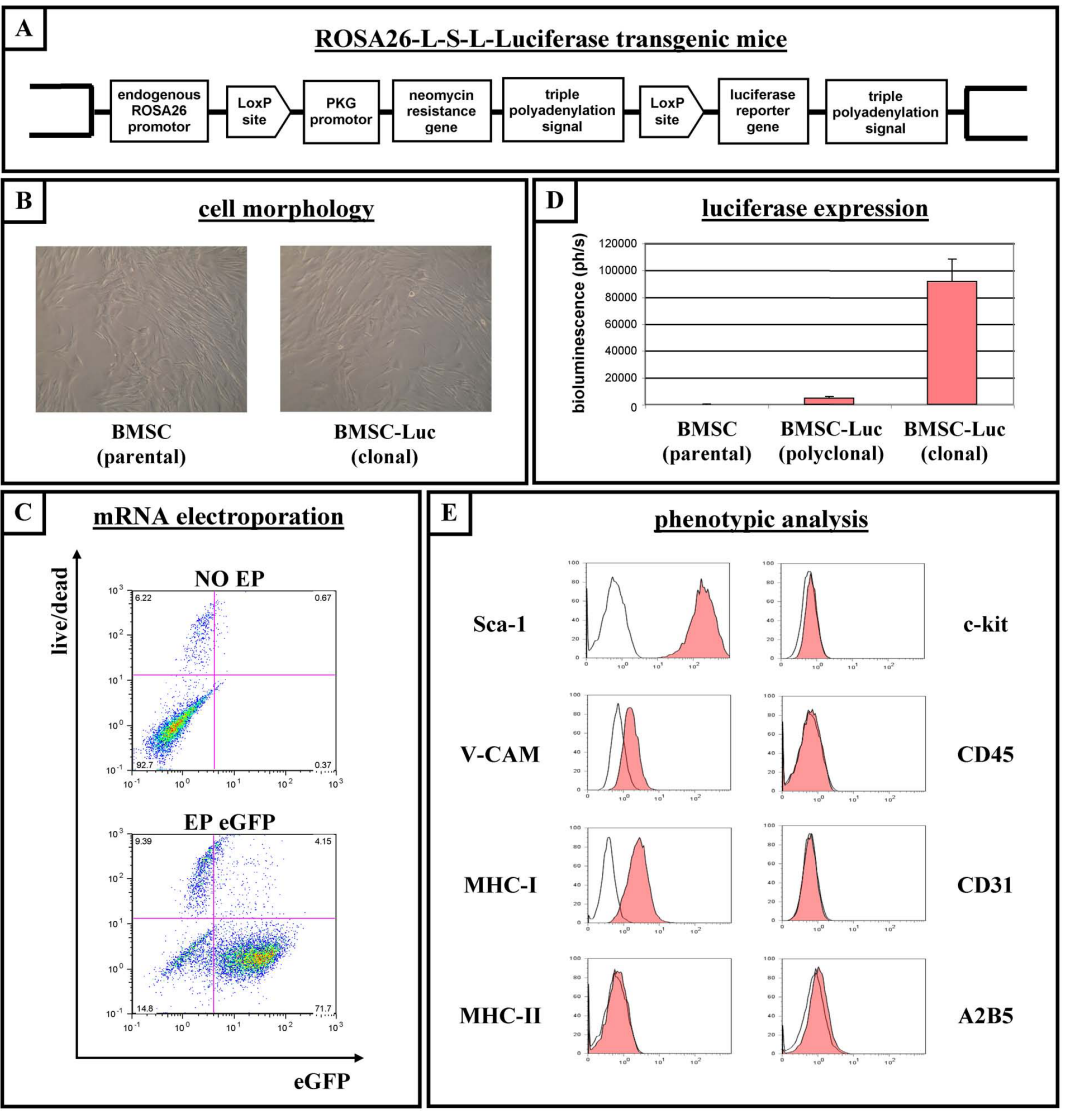
**Culture and characterisation of a clonal luciferase-expressing bone marrow-derived stromal cell line from ROSA26-L-S-L-Luciferase transgenic mice**. (A) Molecular organisation of the ROSA26 locus in ROSA26-L-S-L-Luciferase transgenic mice. (B) Representative pictures of cultured bone marrow-derived stromal cells (BMSC) taken under phase contrast microscopy. Left: unmodified parental BMSC derived from ROSA26-L-S-L-Luciferase transgenic mice (BMSC parental). Right: clonal luciferase-expressing BMSC derived from ROSA26-L-S-L-Luciferase transgenic mice (BMSC-Luc clonal). (C) Parental BMSC derived from ROSA26-L-S-L-Luciferase transgenic mice were non-electroporated (NO EP, upper dot plot) or electroporated with eGFP mRNA (EP eGFP, lower dot plot), and were analyzed by flow cytometry for eGFP fluorescence (x-axis) versus viability (GelRed-staining, y-axis) after 24 hours of culture. The percentage indicated in the lower left quadrant is the number of viable eGFP-negative cells. The percentage indicated in the lower right quadrant is the number of viable eGFP-positive cells. The percentages indicated in the upper left and right quadrant are numbers of non-viable cells. Representative dot plots are shown. (D) *In vitro *luminescence assay on parental BMSC (BMSC parental), on Cre-recombined polyclonal luciferase-expressing BMSC (BMSC-Luc polyclonal), and on Cre-recombined clonal luciferase-expressing BMSC (BMSC-Luc clonal), all derived from ROSA26-L-S-L-Luciferase transgenic mice. (E) Representative flow cytometric analysis showing expression pattern of membrane proteins on clonal BMSC-Luc derived from ROSA26-L-S-L-Luciferase transgenic mice (i.e. expression of Sca-1, V-CAM and MHC-I, but no expression of MHC-II, c-kit, CD45, CD31 and A2B5). Open histograms: control. Filled histograms: specific antibody staining.

ROSA26-L-S-L-Luciferase transgenic mice were originally described as a mouse reporter strain for non-invasive monitoring of *in vivo *Cre-recombination by bioluminescence imaging[20]. In these mice, a luciferase reporter gene, which is preceded by a loxP-flanked neomycin resistance gene, was integrated into the ROSA26 genomic locus by homologous recombination (Figure 1A). In this study, we first attempted to culture bone marrow-derived stromal cells (BMSC) starting from bone marrow of 3-week old ROSA26-L-S-L-Luciferase transgenic mice, as described in detail in the Materials and Methods section. At passage 3, when cultures became homogenous for cells with BMSC morphology (Figure 1B, left picture), cell cultures (n = 3) were analysed by flow cytometry for the presence and absence of typical membrane proteins characteristic for defining murine BMSC [21]. Flow cytometric analysis indicated that the cultured cell populations displayed uniform expression of Sca-1, V-CAM, and MHC-I, without detectable expression of haematopoietic (CD45, c-kit, MHC-II), endothelial (CD31) and neural (A2B5) membrane proteins (data not shown). One parental BMSC line was then chosen ad random and used for further experiments described in this manuscript. In order to allow expression of the luciferase protein in BMSC derived from ROSA26-L-S-L-Luciferase transgenic mice, a "floxed" neomycin resistance cassette needs to be excised by the Cre recombinase protein. We previously described a methodology for efficient Cre-mediated excision of target sequences following electroporation of cells with messenger (m)RNA encoding Cre recombinase [22,23]. Following this strategy, we first evaluated whether murine BMSC were susceptible for mRNA-based gene transfer. Flow cytometric analysis of BMSC electroporated with mRNA encoding the enhanced green fluorescent protein (eGFP) indicated efficient transgene expression in up to 80% of cells at 24 hours post-electroporation (Figure 1C, n = 4). Next, parental BMSC were electroporated with mRNA encoding the Cre recombinase protein in order to activate luciferase expression. A polyclonal luciferase-expressing BMSC line was obtained, which displayed stable expression of luciferase protein for at least 10 passages, as demonstrated by standard *in vitro *luminescence assays (Figure 1D, n = 8). Next, multiple clonal luciferase-expressing BMSC lines were obtained by limiting dilution and screened for high luciferase activity by standard *in vitro *luminescence assay. One clonal line was chosen and used for further characterisation and transplantation experiments described below. This clonal luciferase-expressing BMSC line (further named as BMSC-Luc) was further characterised *in vitro *based on (1) BMSC morphology (Figure 1B, right picture) and immune phenotype (Figure 1E), for which no difference was observed with parental BMSC derived from ROSA26-L-S-L-luciferase transgenic mice, and based on (2) luciferase activity, which was increased as compared to the described polyclonal luciferase-expressing BMSC cells and remained stable over at least 20 passages as demonstrated by standard *in vitro *luminescence assays (Figure 1D, n = 10; and see Additional file 1).

### Survival of luciferase-expressing BMSC derived from ROSA26-L-S-L-Luciferase transgenic mice following implantation in the central nervous system of syngeneic immunocompetent mice

**Figure 2:**
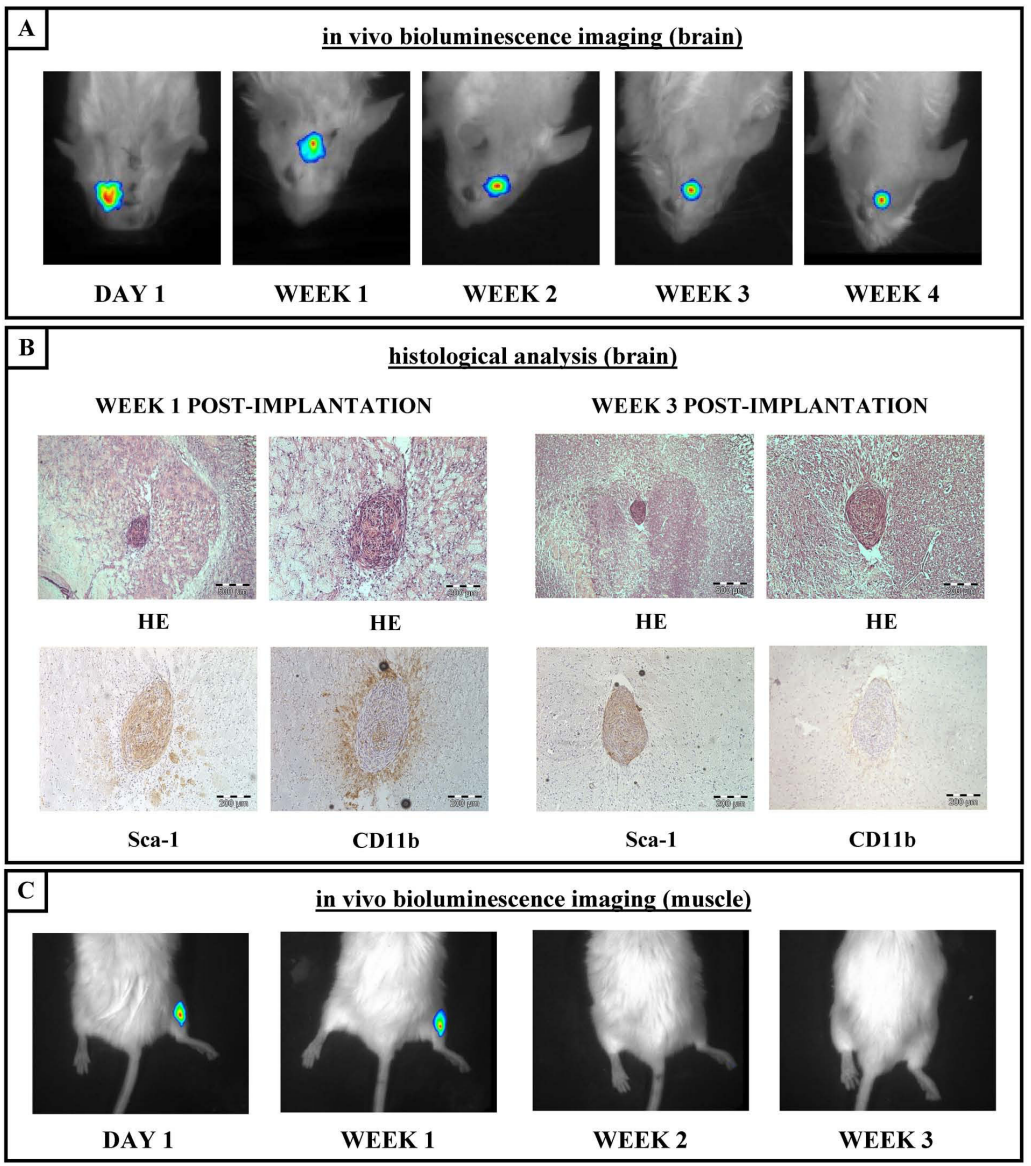
**Survival of luciferase-expressing BMSC derived from ROSA26-L-S-L-Luciferase transgenic mice following implantation in the central nervous system of syngeneic immunocompetent mice**. (A) Representative time course for *in vivo *bioluminescence imaging of clonal BMSC-Luc derived from ROSA26-L-S-L-Luciferase transgenic mice following implantation in the central nervous system of syngeneic immunocompetent mice. (B) Representative histological analysis of clonal BMSC-Luc grafts in the central nervous system of syngeneic immunocompetent mice. Week 1 post-implantation: Upper pictures, haematoxylin-eosin (HE) staining indicating localisation and general appearance of the implantation site. Lower left picture, Sca-1 staining indicating the BMSC origin of the observed cell graft. Lower right picture, CD11b staining indicating the presence of activated microglia surrounding the observed cell graft. Week 3 post-implantation: Upper pictures, HE staining indicating localisation and general appearance of the implantation site. Lower left picture, Sca-1 staining indicating the BMSC origin of the observed cell graft. Lower right picture, CD11b staining indicating the absence of activated microglia surrounding the observed cell graft. All slides were examined using a conventional bright field microscope and digital pictures were taken under magnification as indicated by the scale bars. (C) Representative time course for *in vivo *bioluminescence imaging of clonal BMSC-Luc derived from ROSA26-L-S-L-Luciferase transgenic mice following intramuscular implantation in syngeneic immunocompetent mice.

In order to investigate whether our cultured clonal BMSC-Luc derived from ROSA26-L-S-L-luciferase transgenic mice can survive intrinsically and/or immunologically upon implantation into the central nervous system (CNS) of immune competent mice, 2 × 10^5 ^cells were grafted in the CNS of syngeneic ROSA26-L-S-L-Luciferase mice (n = 28), following procedures described in detail in the Materials and Methods section. In this experimental context, only the luciferase protein produced by the implanted BMSC-Luc can be seen as a potential immunogenic antigen in the CNS. Next, survival of BMSC-Luc implants was monitored by real-time *in vivo *bioluminescence imaging (BLI) until day 1, week 1, week 2, week 3 or week 4 post-implantation. Only mice showing a clear BLI signal on day 1 post-implantation (60%), indicating successful cell implantation in the CNS for BLI imaging, were included for long-term follow-up by BLI. For these mice, a clear BLI signal was detected in 17/17 mice analysed at week 1 post-implantation, in 17/17 mice analysed at week 2 post-implantation, in 6/6 mice analysed at week 3 post-implantation, and in 5/5 mice analysed at week 4 post-implantation. These results suggest both intrinsic and immunological survival of BMSC-Luc cell implants in the CNS of syngeneic immunocompetent mice (Figure 2A), despite potential immunogenicity of the luciferase transgene. In addition, at different time-points post-implantation, randomly chosen animals (both from BLI and non-BLI-group) were sacrificed and dissected brains were analysed for cell graft survival and inflammatory responses. As shown in Figure 2B, cell implants are clearly visible at week 1 and week 3 post-implantation following haematoxylin-eosin (HE) staining of brain slices. These results also validate the observed *in vivo *BLI results described above. Moreover, immunohistochemical staining for Sca-1 clearly identifies the BMSC origin of the cell implants observed (Figure 2B). In addition, we investigated whether the implantation of autologous (syngeneic) BMSC-Luc in the CNS of mice triggers inflammatory responses. For this, brain sections were stained for the presence of CD11b+ activated microglia in the surroundings of grafted BMSC-Luc (Figure 2B). Results indicate that at an early time point post-implantation (week 1) microglial activation does occur, however, this inflammatory response is only temporary as the presence of these CD11b+ activated microglial cells is highly diminished at a later time point post-implantation (week 3). In contrast to the above-described results, intramuscular BMSC-Luc cell implants did not survive beyond week 1 post-implantation as demonstrated by *in vivo *BLI (Figure 2C). For the latter, a clear BLI signal was detected in 8/9 mice analysed at day 1 post-implantation, in 4/7 mice analysed at week 1 post-implantation, in 0/7 mice analysed at week 2 post-implantation and in 0/7 mice analysed at week 3 post-implantation.

### Survival of BMSC genetically modified with multiple reporter genes following implantation in the central nervous system of syngeneic immunocompetent mice

**Figure 3:**
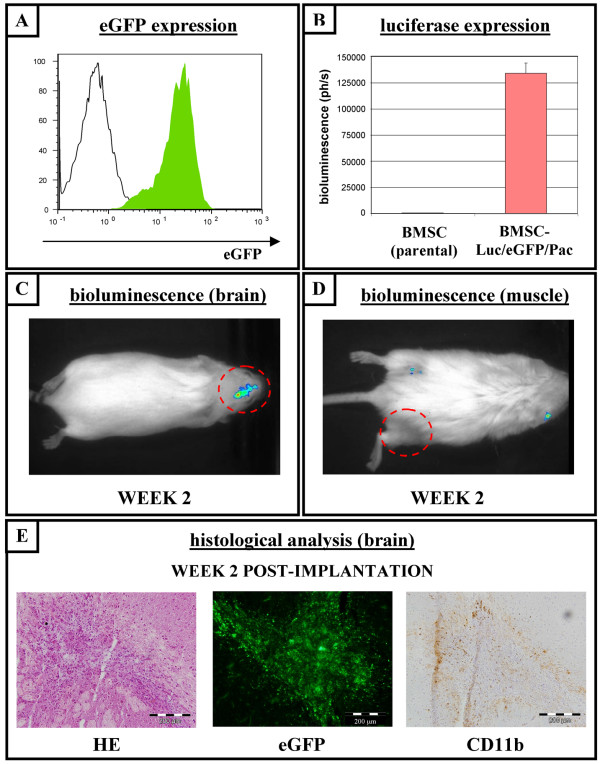
**Survival of BMSC genetically modified with multiple reporter genes following implantation in the central nervous system of syngeneic immunocompetent mice**. (A) Histogram overlay showing a representative flow cytometric analysis of eGFP expression by BMSC expressing the luciferase-, eGFP- and puromycin resistance genes (BMSC-Luc/eGFP/Pac, filled histogram). Parental BMSC were used as negative control (open histogram). (B) *In vitro *luminescence assay on parental BMSC and on clonal BMSC-Luc/eGFP/Pac. (C) *In vivo *real time bioluminescence imaging (BLI) of clonal BMSC-Luc/eGFP/Pac grafts in the central nervous system (CNS) of syngeneic immunocompetent mice at week 2 post-implantation. (D) *In vivo *real time BLI of clonal BMSC-Luc/eGFP/Pac intramuscular grafts in syngeneic immunocompetent mice at week 2 post-injection. (E) Representative histological analysis of clonal BMSC-Luc/eGFP/Pac implants in the CNS of syngeneic immunocompetent mice at week 2 post-implantation. Left picture: haematoxylin-eosin (HE) staining showing general appearance of the cell implantation site. Middle picture: direct eGFP-fluorescence indicating the BMSC-Luc/eGFP/Pac origin of the observed cell implant. Right picture: CD11b staining indicating a limited number of microglia surrounding the observed cell graft. All slides were examined using a conventional bright field microscope and digital pictures were taken under magnification as indicated by the scale bars.

In order to investigate whether the immunological survival of BMSC-Luc in the CNS of syngeneic immunocompetent mice was due to low immunogenicity of the luciferase protein in the CNS, we introduced additional xenogeneic reporter genes into our cultured BMSC-Luc. For this, BMSC-Luc were transduced with a lentivirus encoding the enhanced green fluorescence protein (eGFP) and the puromycin resistance gene (Pac). Following puromycin selection and single clone selection, a clonal Luciferase-, EGFP- and Pac-expressing BMSC line was obtained (further named as BMSC-Luc/eGFP/Pac). Flowcytometric analysis of BMSC-Luc/eGFP/Pac demonstated eGFP transgene expression in > 95% of the cells (Figure 3A), which remained stable in culture for at least 15 passages. In addition, phenotypical properties (data not shown) and luciferase activity (Figure 3B, n = 3) were not influenced following lentiviral-transduction of BMSC-Luc. Next, in order to investigate the *in vivo *immunological survival of BMSC genetically-modified with multiple reporter genes in immunocompetent mice, 2 × 10^5 ^BMSC-Luc/eGFP/Pac were implanted in the CNS of syngeneic ROSA26-L-S-L-luciferase transgenic mice (n = 8). Survival of grafted BMSC-Luc/eGFP/Pac was then monitored by *in vivo *BLI on day 1, week 1 and week 2 post-implantation (Figure 3C). A clear BLI signal was observed in 8/8 mice analysed at day 1 post-implantation, in 6/8 mice analysed at week 1 post-implantation and in 6/6 mice analysed at week 2 post-implantation. In addition, histological analysis (Figure 3E, n = 2) at week 2 post-implantation confirmed: (I) the presence of eGFP-expressing BMSC-Luc/eGFP/Pac implants, and (II) a limited number of activated CD11b+ microglia surrounding BMSC-Luc/eGFP/Pac implants, indicating survival of BMSC-Luc/eGFP/Pac cell implants in the CNS of syngeneic immunocompetent mice. In contrast, intramuscular BMSC-Luc/eGFP/Pac implants did not survive beyond week 1 post-implantation (Figure 3D) as demonstrated by *in vivo *BLI. In these experiments, a clear BLI signal was detected in 4/4 mice at day 1 post-implantation, in 3/4 mice at week 1 post-implantation and in 0/4 mice at week 2 post-implantation.

### Induction of BMSC-specific CD8+ T-cell responses following intramuscular, but not intracerebral, cell implantation in syngeneic immunocompetent mice

**Figure 4:**
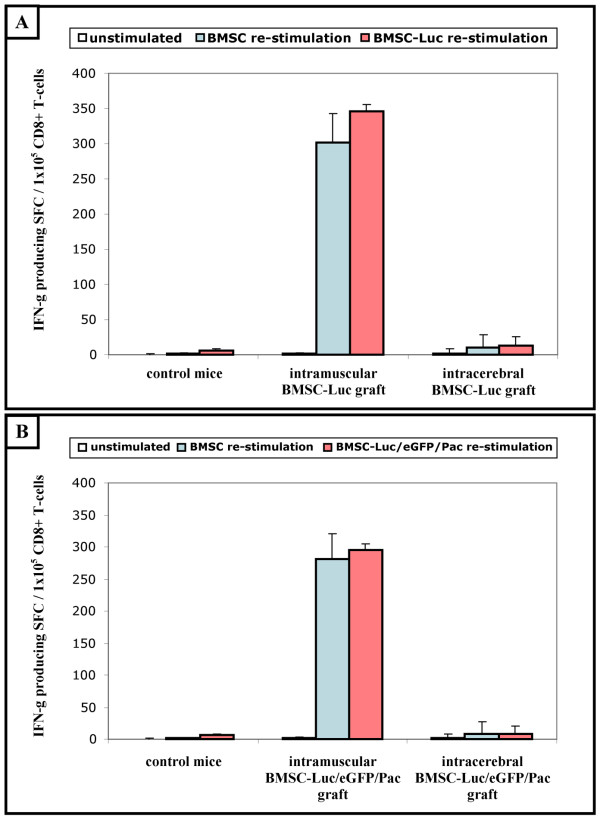
**Induction of BMSC-specific CD8+ T-cell responses following intramuscular, but not intracerebral, cell implantation in syngeneic immunocompetent mice**. (A) Spleen CD8+ T-cells (1 × 10^5 ^cells/well) from non-transplanted ROSA26-L-S-L-Luciferase mice (control mice, n = 4) and ROSA26-L-S-L-Luciferase mice with BMSC-Luc implants, either intramuscularly (intramuscular BMSC-Luc graft, n = 5) or intracerebrally (intracerebral BMSC-Luc graft, n = 6), were cultured in quadruplet in an IFN-γ ELISPOT assay alone (unstimulated), with addition of parental BMSC (BMSC re-stimulation) or with addition of BMSC-Luc (BMSC-Luc re-stimulation) (1 × 10^4 ^cells/well, ratio 10:1). Data are expressed as the mean number of IFN-γ spot forming cells (SFC)/1 × 10^5 ^CD8+ T-cells for each experimental group. (B) Spleen CD8+ T-cells (1 × 10^5 ^cells/well) from non-transplanted ROSA26-L-S-L-Luciferase mice (control mice, n = 4) and ROSA26-L-S-L-Luciferase mice with BMSC-Luc/eGFP/Pac implants, either intramuscularly (intramuscular BMSC-Luc/eGFP/Pac graft, n = 5) or intracerebrally (intracerebral BMSC-Luc/eGFP/Pac graft, n = 6), were cultured in quadruplet in an IFN-γ ELISPOT assay alone (unstimulated), with addition of parental BMSC (BMSC re-stimulation) or with addition of BMSC-Luc/eGFP/Pac (BMSC-Luc/eGFP/Pac re-stimulation) (1 × 10^4 ^cells/well, ratio 10:1). Data are expressed as the mean number of IFN-γ spot forming cells (SFC)/1 × 10^5 ^CD8+ T-cells for each experimental group.

In order to investigate whether the non-survival of intramuscular BMSC-Luc and BMSC-Luc/eGFP/Pac cell implants was mediated by the host's immune system, we evaluated the presence of reporter gene-specific interferon (IFN)-γ-producing CD8+ T-cells by ELISPOT analysis. For this, spleen CD8+ T-cells were isolated from control mice and from BMSC-Luc or BMSC-Luc/eGFP/Pac cell implanted mice (both intramuscular and intracerebral implants) at week 2 post-implantation. Then, isolated CD8+ T-cells were un-stimulated, re-stimulated with parental BMSC, or re-stimulated with BMSC-Luc or BMSC-Luc/eGFP/Pac in an IFN-γ ELISPOT assay (Figure 4). Interestingly, no significant number of IFN-γ-producing CD8+ T-cells directed against BMSC-Luc (figure 4A; BMSC-Luc re-stimulation – control mice (n = 4) versus intracerebral BMSC-Luc graft (n = 6) – p = 0.07) or BMSC-Luc/eGFP/Pac (figure 4B; BMSC-Luc/eGFP/Pac re-stimulation – control mice (n = 4) versus intracerebral BMSC-Luc/eGFP/Pac graft (n = 6) – p = 0.32) cell implants was detected upon intracerebral cell implantation. However, a large number of reactive IFN-γ-producing CD8+ T-cells directed against BMSC-Luc (figure 4A; BMSC-Luc re-stimulation – control mice versus intramuscular BMSC-Luc graft (n = 5) – p < 0.001) or BMSC-Luc/eGFP/Pac (figure 4B; BMSC-Luc/eGFP/Pac re-stimulation – control mice versus intramuscular BMSC-Luc/eGFP/Pac graft (n = 5) – p < 0.001) was observed upon intramuscular cell implantation. These results demonstrate that BMSC-Luc and BMSC-Luc/eGFP/Pac specific IFN-γ-producing T-cells are efficiently induced upon intramuscular cell implantation, but not upon intracerebral cell implantation, indicating potential immunogenicity of our reporter gene-modified BMSC for the peripheral immune system, but not for the CNS immune system. In an attempt to demonstrate that the observed IFN-γ-producing T-cells were specific for Luciferase, eGFP or Pac, isolated CD8+ T-cells were also re-stimulated with parental BMSC. Surprisingly, CD8+ T-cells isolated from intramuscular BMSC-Luc or BMSC-Luc/eGFP/Pac cell implanted mice were equally reactive against parental non-modified BMSC (figure 4A; intramuscular BMSC-Luc graft – BMSC re-stimulation versus BMSC-Luc re-stimulation – p = 0.06) (figure 4B; intramuscular BMSC-Luc/eGFP/Pac graft – BMSC re-stimulation versus BMSC-Luc/eGFP/Pac re-stimulation – p = 0.26) The latter indicates that the non-survival of intramuscular BMSC-Luc or BMSC-Luc/eGFP/Pac cell implants – in our experimental model – is not mediated by reporter gene-specific T-cells, although it is unclear at the moment which antigens were responsible for initiating T-cell activation against our cultured BMSC.

## Discussion

In many pre-clinical cell therapy studies, reporter gene-assisted imaging of cellular implants in the CNS and potential reporter gene and/or cell-based immunogenicity, still remain challenging research topics. In this study, we first aimed to investigate whether luciferase-expressing bone marrow-derived stromal cells (BMSC), derived from ROSA26-L-S-L-Luciferase transgenic mice, can be implanted and survive in the CNS of immunocompetent syngeneic luciferase-negative ROSA26-L-S-L-Luciferase transgenic mice, despite the potential immunogenicity of the luciferase protein [19,20]. The choice of ROSA26-L-S-L-Luciferase transgenic mice for performing these experiments has two reasons. First, we assumed that the epigenetic stability of luciferase expression would be much higher when cell populations were derived from a well-characterised luciferase-expressing transgenic mouse strain, as compared to *ex vivo *transgenesis using plasmid DNA or viruses [11,26]. Second, Cre recombination in cells derived from ROSA26-L-S-L-Luciferase transgenic mice allows removal of a floxed neomycin resistance gene (Figure 1A), resulting in luciferase protein expression without additional selection markers. Following this strategy, i.e. derivation of cell populations from ROSA26-L-S-L-Luciferase transgenic mice followed by Cre-recombination in order to activate luciferase expression, autologous transplantation experiments can be performed in syngeneic luciferase-negative ROSA26-L-S-L-Luciferase transgenic mice with only the luciferase protein as potential immunogen. In this context, we derived BMSC cultures from ROSA26-L-S-L-Luciferase transgenic mice and characterised these BMSC populations as described by Peister et al [21]. Immunophenotypic analysis (Figure 1E) clearly demonstrated the uniform expression of mesenchymal markers (Sca-1 and V-CAM) without detectable expression of endothelial (CD31), haematopoietic (c-kit, CD45 and MHC-II) or neural (A2B5) markers.

Next, in order to allow expression of the luciferase protein in BMSC derived from ROSA26-L-S-L-Luciferase transgenic mice, a floxed neomycine resistance cassette needs to be excised by the Cre recombinase protein. We previously described a non-viral non-DNA gene transfer methodology for highly efficient protein expression in a variety of cell types, including human BMSC, based on electroporation of messenger RNA [27-30]. In this study, following these previous reports, we also describe for the first time highly efficient mRNA-based gene transfer in murine BMSC using the enhanced green fluorescent protein (eGFP) reporter gene (Figure 1C). The latter is of importance when transient protein expression is desired and introduction of DNA sequences (either by plasmid DNA or viruses) should be avoided [31,32]. Next, our cultured BMSC populations were electroporated with mRNA encoding the Cre recombinase protein, following previously described procedures [22,23]. Although luciferase expression was induced (Figure 1D, BMSC-Luc polyclonal), the culture of a clonal luciferase-expressing BMSC was necessary in order to obtain a pure population expressing high levels of the luciferase protein (Figure 1D, BMSC-Luc clonal). The fact that recombination efficiency was rather low in cultured BMSC following electroporation with Cre recombinase mRNA, despite the observation that electroporation with EGFP mRNA resulted in high levels of transfection efficiency, can be ascribed to variations in Cre recombinase activity in different cell types (published and unpublished data) [22,23].

In our transplantation model, i.e. autologous implantation of BMSC-luc derived from ROSA26-L-S-L-Luciferase transgenic mice in the CNS of syngeneic luciferase-negative ROSA26-L-S-L-Luciferase transgenic mice, we routinely transplant 2 × 10^5 ^cells in order to obtain a clear signal for in vivo bioluminescence imaging (BLI). Further experiments revealed a minimum of 5 × 10^4 ^cells to be required for obtaining a minimum signal above background (data not shown). However, this detection limit might be different when using BMSC derived from another luciferase-expressing transgenic mouse or following lentiviral transduction with the luciferase reporter protein. Following cell transplantation in this model, we did not observe immune-mediated rejection of BMSC-Luc implants in the CNS during a follow-up period of 3-4 weeks by real-time BLI (Figure 2A), while intramuscular BMSC-Luc implants did not survive during the same follow-up period (Figure 2C). Also, when the same BMSC population was implanted in the CNS of immunocompetent allogeneic C57/BL6 mice (see Additional file 2) or when C57/BL6 BMSC were implanted in the CNS of immunocompetent ROSA26-L-S-L-Luciferase transgenic mice (data not shown), no survival of grafted cells was observed during the same follow-up period. These results suggest that BMSC-Luc derived from ROSA26-L-S-L-Luciferase transgenic mice can indeed survive immunologically in the CNS of immunocompetent luciferase-negative ROSA26-L-S-L-Luciferase transgenic mice, despite the potential immunogenicity of the luciferase protein. In addition, during the observation period of 3-4 weeks, we did not observe a significant increase of in vivo bioluminescence signal over time. The latter, although further investigation will be needed (e.g. quantitative analysis), might become a tool to exclude tumour formation following cell implantation [12].

In order to further investigate the tolerogenic properties of the CNS with regard to reporter gene-modified BMSC implants, we further genetically engineered our BMSC-Luc cells using a lentivirus encoding eGFP and the puromycin resistance gene (Figure 3A). Following transplantation of these BMSC-Luc/eGFP/Pac in the CNS of syngeneic immunocompetent mice, a similar degree of cell survival was observed as compared to BMSC-Luc implants (Figure 3C and 3E). Again, no cell survival was observed upon intramuscular BMSC-Luc/eGFP/Pac implantation. These results demonstrate that reporter gene-modified BMSC can survive immunologicaly in the CNS of syngeneic immunocompetent mice. Currently, we do not know why expression of reporter proteins (in this study Luc, eGFP and Pac), which are from an immunological point of view a foreign antigens, are tolerated in the CNS. Several explanations can be hypothesised for this: (1) some cell populations, among them BMSC, have been ascribed immune modulatory properties [33], or (2) immune surveillance mechanisms in the CNS are not properly activated [34], both possibly leading to immunological acceptance of the neo-expressed reporter proteins in the CNS. In this context, we investigated whether inflammatory responses occur following cell implantation in the CNS. Although histological analysis of cell-implanted brains indicated the presence of activated CD11b+ microglial cells surrounding the cell graft at week 1 post-implantation, the presence of these CNS immune cells was highly diminished by week 3 post-implantation, indicating immunological acceptance of autologous BMSC-Luc (Figure 2B) or BMSC-Luc/eGFP/Pac (Figure 3E). However, the observed immune tolerance of the CNS for reporter gene-modified BMSC does not imply an absolute immune tolerance of the CNS. In contrast, allogeneic cell implantation in the CNS of immunocompetent mice leads to a sustained activation of microglia and rejection of cell implants by week 2–4 post-implantation (see Additional file 2).

Finally, we aimed to investigate whether the non-survival of intramuscular BMSC-Luc and BMSC-Luc/eGFP/Pac cell implants was mediated by the host's immune system. Although the presence of reactive IFN-γ-producing CD8+ T-cells was clearly demonstrated following intramuscular, but not intracerebral, BMSC-Luc and BMSC-Luc/eGFP/Pac cell implantation (Figure 4), surprisingly these immune reactive T-cell response were not specific for the introduced reporter genes. Although further research will be needed to elucidate the specificity of the induced BMSC-specific IFN-γ-producing CD8+ T-cells, several explanations can be hypothesised for this: (1) due to the use of fetal calf serum and horse serum for *in vitro *BMSC expansion, xenogeneic serum components (eg. glycolipids) might have induced cellular immunogenicity, or (2) cell culture induced genomic alterations might have resulted in the expression of highly immunogenic neo-antigens, both possibly leading to immunological rejection of our BMSC cultures following intramuscular cell implantation.

## Conclusion

While many cell transplantation studies are currently performed under immunosuppressive therapy or in immune-deficient mice, clinical applications of cell therapy will most likely have to deal with immunocompetent patients. In this study, we demonstrate that reporter gene-modified BMSC derived from ROSA26-L-S-L-Luciferase transgenic mice are immune-tolerated upon cell implantation in the CNS of syngeneic immunocompetent mice. The proposed research model thus provides a powerful tool for studying survival and localisation of autologous BMSC implants in the central nervous system of syngeneic mice by real-time bioluminescence imaging and/or histological analysis in the absence of immunosuppressive therapy.

## Authors' contributions

IB carried out cell culture, flow cytometry, cell transplantations, bioluminescence imaging, histological analysis, ELISPOT analysis, data collection, data interpretation and drafted the manuscript. NDV carried out cell culture, cell transplantations and bioluminescence imaging, BT carried out histological analysis, ELISPOT analysis, and assisted with data interpretation, JV assisted with bioluminescence imaging, KR assisted with cell culture, JD carried out mRNA preparation, cell transfections and assisted with cell culture and flow cytometry. AI carried out lentiviral transduction experiments. VFIVT acquired funding. SC acquired funding and assisted in evaluation of histological analysis. HG acquired funding. PGJ acquired funding and assisted in study design. VB provided expertise and support for lentiviral transduction experiments. DY provided support for animal experiments. EVM provided support for histological analysis. ZB acquired funding and assisted in study design and data interpretation. AVDL acquired funding and assisted in study design and data interpretation. PP acquired funding, carried out study design, data collection, data interpretation and drafted the manuscript. All authors have read and approved the manuscript.

## Supplementary Material

Additional file 1 ***In vitro *****bioluminescence of cultured luciferase-expressing bone marrow-derived stromal cells using the Biospace*****in vivo *****bioluminescence camera**. Additional data showing detection sensitivity of *in vitro *bioluminescence by luciferase-expressing bone marrow-derived stromal cells using the Biospace *in vivo *bioluminescence camera. Click here for file

Additional file 2 **Histological analysis of luciferase-expressing ROSA26-L-S-L-Luc bone marrow-derived stromal cells following allogeneic transplantation in C57BL/6 mice**. Additional data showing immunological rejection of luciferase-expressing ROSA26-L-S-L-Luc bone marrow-derived stromal cells following allogeneic transplantation in C57BL/6 mice. Click here for file
