# Supplementary material for: Reporter gene-expressing bone marrow-derived stromal cells are immune-tolerated following implantation in the central nervous system of syngeneic immunocompetent mice
Source: BMC Biotechnol. 2009 Jan 7;9:1. doi: 10.1186/1472-6750-9-1 (PMC2630974; doi:10.1186/1472-6750-9-1)
Supplement: Additional file 1 — In vitro bioluminescence of cultured luciferase-expressing bone marrow-derived stromal cells using the Biospacein vivo bioluminescence camera. Additional data showing detection sensitivity of in vitro bioluminescence by luciferase-expressing bone marrow-derived stromal cells using the Biospace in vivo bioluminescence camera. [file 1472-6750-9-1-S1.pdf]

In vitro bioluminescence of cultured luciferase-expressing bone marrow-derived stromal cells using the Biospace in vivo bioluminescence camera.

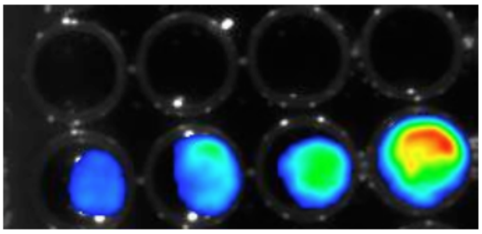

BMSC

BMSC-Luc

Upper row: negative control showing bioluminescence signal of luciferase-negative ROSA26-L-S-L-Luc bone marrow-derived stromal cells (BMSC) per well ranging from 2,5 x 10^5 (left) to 10 x 10^5 (right) cells after administration of luciferin (1,5 mg). **Lower row:** image showing bioluminescence signal of luciferase-expressing ROSA26-L-S-L-Luc bone marrow-derived stromal cells (BMSC-Luc) per well ranging from 2,5 x 10^5 (left) to 10 x 10^5 (right) cells after administration of luciferin (1,5 mg). The most intense bioluminescence signal detected is shown in red, while the weakest signal is shown in blue.
