# Supplementary material for: Reporter gene-expressing bone marrow-derived stromal cells are immune-tolerated following implantation in the central nervous system of syngeneic immunocompetent mice
Source: BMC Biotechnol. 2009 Jan 7;9:1. doi: 10.1186/1472-6750-9-1 (PMC2630974; doi:10.1186/1472-6750-9-1)
Supplement: Additional file 2 — Histological analysis of luciferase-expressing ROSA26-L-S-L-Luc bone marrow-derived stromal cells following allogeneic transplantation in C57BL/6 mice. Additional data showing immunological rejection of luciferase-expressing ROSA26-L-S-L-Luc bone marrow-derived stromal cells following allogeneic transplantation in C57BL/6 mice. [file 1472-6750-9-1-S2.pdf]

Histological analysis of luciferase-expressing ROSA26-L-S-L-Luc bone marrow-derived stromal cells following allogeneic transplantation in C57BL/6 mice.

WEEK 3 POST-IMPLANTATION

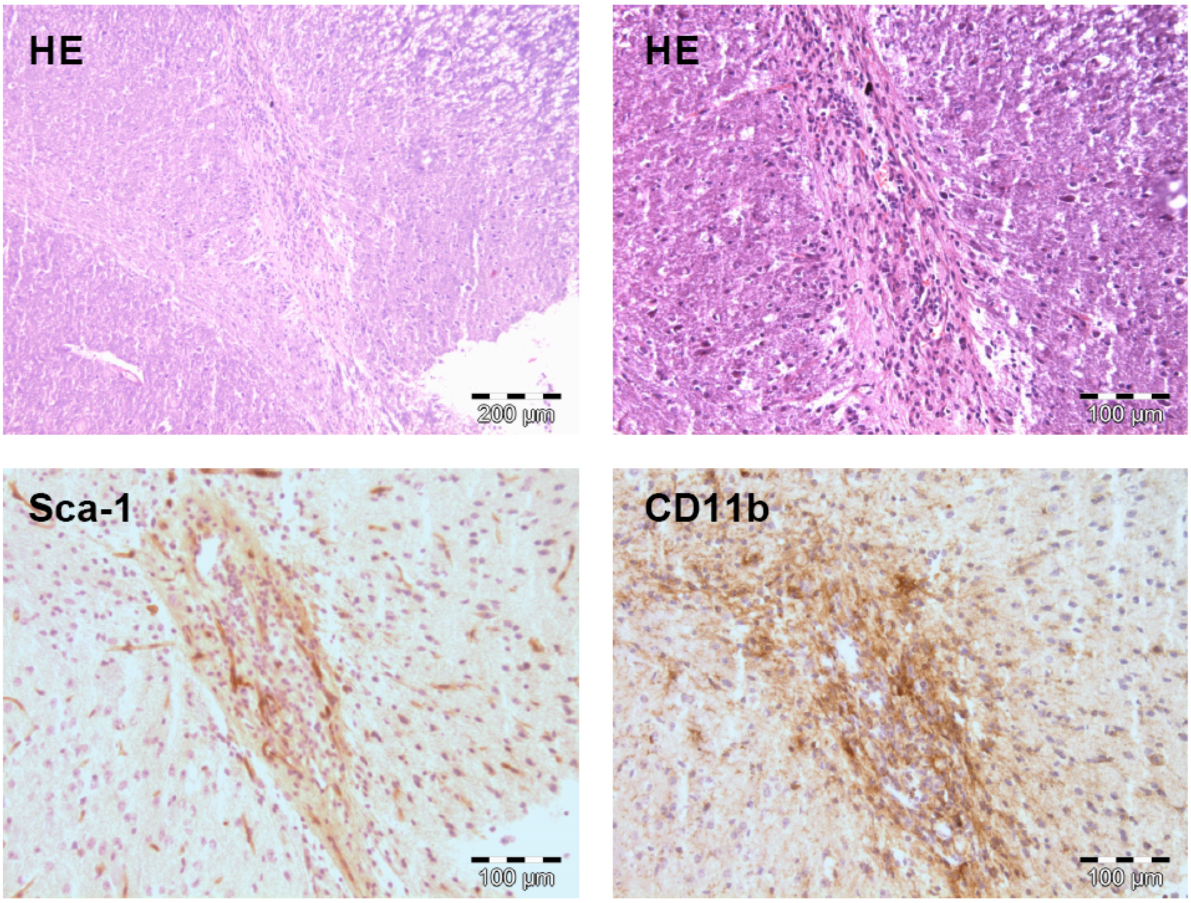

Upper pictures: haematoxylin-eosin (HE) staining indicating localisation and general appearance of the implantation site. **Lower left picture:** diminished Sca-1 staining indicating rejection of implanted allogeneic BMSC-Luc. **Lower right picture:** CD11b staining indicating the presence of activated microglia extensively surrounding and invading the graft site. All slides were examined using a conventional bright field microscope and digital pictures were taken under magnification as indicated by the scale bars.
